# Embracing complexity: An opportunity for developmental evaluation

**DOI:** 10.1017/cts.2026.10767

**Published:** 2026-06-05

**Authors:** Brian J. Biroscak, Peter S. Hovmand

**Affiliations:** Center for Community Health Integration, Case Western Reserve University School of Medicine, Cleveland, OH, USA

**Keywords:** Developmental evaluation, clinical and translational science, complexity, systems thinking, innovation development

## Introduction

In the rapidly evolving field of clinical and translational science (CTS), evaluating the effectiveness of innovations is crucial for informing decision-making and ensuring efficient use of resources [1]. Traditional evaluation approaches, while valuable, often fall short when dealing with the complexities and uncertainties inherent in translational research projects [2]. These methods typically assume stable, predictable conditions which are rarely present in dynamic research environments [3].

Our position is that developmental evaluation (DE) should be more widely practiced among CTS innovators and evaluators. Many CTS innovations are complex and multifaceted, requiring navigation across various scientific paradigms, institutional cultures, and regulatory environments [4]. Traditional evaluations, which typically follow a linear design-implement-evaluate model [5], struggle to adapt to the fluid and often unpredictable nature of such innovation settings (Table 1). This rigidity can hinder the ability to accurately assess and guide the development of interventions, impacting their success and sustainability [6].

**Table 1.** Comparison of traditional and developmental evaluation approaches

Aspect	Developmental evaluation	Traditional evaluation
Purpose	Supports ongoing development and adaptation	Assesses effectiveness of established interventions
Approach	Iterative and flexible; adapts to contexts	Linear and fixed; follows a set plan
Feedback Mechanism	Continuous, real-time feedback	Periodic feedback at project milestones
Focus	Learning and adaptation during project lifecycle	Measuring outcomes against fixed criteria
Suitability	Ideal for complex, uncertain, and evolving environments (e.g., pilot implementation of a new digital health tool in a community setting)	Best for stable, predictable conditions (e.g., Phase III clinical trials)
Stakeholder Involvement	Active and ongoing participation from stakeholders	Primarily involved in initial planning and reporting
Role of the Evaluator	Embedded “critical friend” who supports learning and adaptation in real time	Independent observer who assesses outcomes after the fact
Decision-making Timing	Real-time and iterative; informs ongoing decisions throughout the project	End-of-cycle or milestone-based; decisions follow formal evaluation reports
Data Usage	Fed back continuously into program design and adaptation	Used to judge fidelity to the original plan or success against fixed criteria

To address these challenges, DE offers a more flexible and adaptive orientation. Defined as an evaluation approach that “supports innovation *development* to guide adaptation to emergent and dynamic realities in complex environments [7],” it is particularly suited for contexts where uncertainty and rapid change prevail [8]. In addition to informing needed innovation *improvements*, DE facilitates the development of interventions, assisting innovators in navigating and adapting to the evolving and complex realities of dynamic environments.

By integrating DE methods, researchers and evaluators can foster continuous feedback and learning, adapting strategies as innovations and circumstances change. Such an approach not only improves the relevance and effectiveness of evaluation practices but also supports the iterative development of innovations in real time [9]. By embracing this opportunity, translational researchers can better navigate the intricate landscape of modern CTS innovations, ensuring interventions are effective and responsive to emergent needs.

## The state of developmental evaluation in translational science

Within the multidisciplinary landscape of CTS, the complexity and inherent uncertainty of innovations necessitate evaluation methods that go beyond the constraints of traditional approaches. Despite the growing recognition of these challenges [10], a systematic examination of published CTS research reveals very few DE examples [11–13]. The limited references to DE in key journals highlight an opportunity for the field to embrace more flexible and responsive approaches. Equally important is to situate DE within the broader landscape of evaluation frameworks that have emerged from implementation science.

Implementation science has produced a rich array of frameworks designed to help innovators navigate complex, real-world environments. The Consolidated Framework for Implementation Research (CFIR) provides a well-validated multilevel structure for identifying determinants of implementation success [14]. The dynamic adaptation process (DAP) explicitly acknowledges that interventions must be tailored to local context during rollout [15]. Design for dissemination and sustainability (D&S) embeds scalability considerations into program development from the outset [16]. These frameworks represent genuine advances over purely linear evaluation approaches: they are context-sensitive, stakeholder-inclusive, and increasingly used across CTS settings. Their limitations, however, are equally instructive. CFIR and DAP are most useful once an interventions’ core logic and components are sufficiently defined to be assessed against contextual determinants. Similarly, D&S presupposes a mature innovation whose dissemination pathway can be mapped. In each case, the framework assumes the “what” is known; the question is “how” to implement, adapt, and sustain it.

Traditional evaluation methods, designed for stable and standardized interventions, struggle to accurately assess the complex, context-dependent nature of novel clinical innovations. The same limitation applies to many implementation science frameworks when applied prematurely. See Table 1 for a comparison of these divergent approaches. Relying solely on these frameworks risks misrepresenting the true value and scalability of promising new technologies.

The translational process involves navigating between different scientific paradigms and the bureaucracy of large healthcare systems [17]. These factors contribute to a landscape where conventional evaluation approaches, which rely on predetermined outcomes and fixed implementation pathways, prove inadequate. In comparison, DE offers an adaptive framework tailored to meet the demands of complex and evolving environments – particularly during the innovation-development phase before a stable intervention exists to implement [7].

CTS innovations often emerge through iterative processes where the intervention and implementation strategy evolve simultaneously, which increases the number of potential roadblocks. As noted by Austin [18]. “All translational science projects must develop a technology or insight or paradigm to improve the efficiency or effectiveness of a rate-limiting translational roadblock, demonstrate its utility in achieving that improvement in one or more use cases, and then actively disseminate these improvements” (p. 1634). By fostering a continuous feedback loop, DE enables researchers and evaluators to refine interventions iteratively, thus producing the stable, well-characterized innovation that implementation science frameworks then require.

This approach not only supports the real-time adaptation of strategies but also enhances the relevance and applicability of research findings. The multi-stakeholder nature of many CTS innovations creates additional complexity. To date, though, DE and other similarly rigorous evaluation approaches are not part of team science discourse across translational science [19]. Team science and translational science partnerships require continuous adaptation and learning, precisely the conditions where DE excels.

Furthermore, DE is fully compatible with systems thinking – one of the distinguishing skills of translational scientists [20]. At the field level, greater use of robust evaluation approaches such as DE has the potential to improve the utility of evaluation findings, which can then speed clinical innovation and enable the use of adaptive data collection and monitoring, all of which would stimulate interest and implementation of robust evaluation. This creates a virtuous feedback loop: improved evaluation leads to faster clinical innovation and enables the use of adaptive data collection, which in turn stimulates broader interest in robust, adaptive evaluation practices.

## A case illustration: the smart innovation program

The Systems Marketing Analysis for Research Translation (SMART) innovation program, funded through a five-year NIH RC2 award at the Case Western Reserve University Clinical and Translational Sciences Collaborative (CTSC) of Northern Ohio, offers a concrete example of DE in practice. SMART was originally designed to provide a menu of social marketing research services to teams engaged in community-based translational work – helping innovators better understand the markets and systems surrounding their innovations. From the outset, the program operated in genuinely emerging territory: no established example existed for delivering systematic social marketing analyses to teams at the level of a clinical translational sciences award hub, and the scope of services to be offered remained an open question.

The limitations of that original design became apparent through the team’s ongoing internal reflection during Year 3 (July 2025–June 2026). Two related challenges surfaced. First, delivering a full menu of social marketing services to each participating team on an individualized basis proved difficult to operationalize at scale. Second, and more generatively, a Co-Investigator raised during a project team meeting that no program analogous to the NIH NCATS I-Corps existed specifically for community-based innovators – and that adapting the I-Corps cohort model for this program could address both the resource challenge and an unmet need in the field. The I-Corps program also carried an evidence base supporting its effectiveness, even if that evidence had been developed with different participant populations.

Rather than treating this as a deviation from the original plan, the SMART team – drawing on prior experience with DE – recognized it as precisely the kind of adaptive signal that DE is designed to surface and act upon. The pivot was neither reactive nor ad hoc; it reflected a structure process of learning and adjustment grounded in real-time feedback from team members closest to implementation. The result was a more feasible, evidence-informed program model better suited to the needs of community-based innovation team members.

This experience illustrates what DE makes possible: not the abandonment of rigor, but the application of rigor to the right question at the right moment. The SMART pivot was not a failure of the original plan – it was the original plan working as it should.

## Conclusion and next steps

We acknowledge that DE is not universally appropriate for all CTS situations. It is most valuable when innovations are genuinely emergent and adaptive, rather than when testing well-established interventions with known parameters. For stable interventions requiring rigorous efficacy testing, traditional evaluation research methods such as randomized controlled trials remain the gold standard.

Detractors may argue that DE lacks methodological rigor. However, this perspective conflates appropriateness with rigor. DE employs systematic data collection and analysis but applies them to different questions. Rather than asking “Does it work?” DE asks, “What is working, for whom, under what conditions, and how can we enhance it?” This is methodologically rigorous and particularly relevant during innovation development phases.

Additionally, some may question whether DE findings are generalizable. While DE produces context-specific insights, these insights inform broader principles about innovation development processes that can guide similar efforts elsewhere. The goal is not statistical generalization but rather analytical generalization of developmental principles and processes.

We call on the JCTS readership to embrace DE as a complement to traditional evaluation approaches. Practically, this means:

- Recognizing when innovations are genuinely emergent;
- Incorporating developmental phases into grant proposals and evaluation plans;
- Building capacity in DE methods among CTS evaluators;
- Sharing experiences using DE to build the evidence base for its application.

As CTS innovations become increasingly complex and collaborative, evaluation approaches must evolve accordingly. By adopting DE more widely, we can better support innovation development, improve resource allocation, and accelerate the translation of research into practice for improved population health outcomes.
